# A Curious Variety of Grippe

**Published:** 1897-02

**Authors:** Frederic Preston

**Affiliations:** Chester, Pa.


### A CURIOUS VARIETY OF GRIPPE.

FREDERIC PRESTON, M. D., CHESTER, PA.

(Translated from *La Semaine Medicale* of December 16th, 1896.)

A disease, the nature of which remains undetermined, but which, from descriptions given, presents more or less the symptoms of a nervous grippe of an unusual nature, is epidemic in the little village of Aransa (province of Lérida), Spain, situate on the southern slope of the Eastern Pyrenees, near the Valley of Andora, at 1,500 meters above sea level, in a very salubrious climate.

The epidemic appeared in June last, and within four days 100 of the 300 inhabitants of the village were attacked.

The principal symptoms of the affection are extreme restlessness and piercing pains at the epigastrium, prostration, and sleeplessness. Temperature, respiratory and digestive organs remain normal. Relapse very frequent. During a certain time adults only were attacked; but since August infants also became victims; the disease presenting symptoms resembling tetanus and meningitis, and resulting, frequently, fatally. During this time the virulence of attacks was somewhat diminished in adult cases, some persons being completely re-established in health, while others still continued to show the symptoms in milder form. During the month of August there was a recrudescence of the disease, and the affection took on a graver character. *Cases of sudden death were noted when the victim was tranquilly conversing, or pursuing his ordinary occupation, or even during sleep.* Others were seized with violent convulsions, followed by hemiplegia, and succumbed after a long and painful illness.

All the means employed against this bizarre affection were powerless, with the exception of change of residence. It is only necessary to quit the village, and inhabit a neighboring locality, to be rapidly cured. But on returning to the contaminated village the illness returns at once.

The medical inquest instituted to establish the nature of the infection, has given no results, except it be to establish the fact that the disease is *not* propagated even in the immediate neighborhood, and that the subjects removed from Aransa were cured, only to relapse when returning to the village.

It may be added that at this date (December 16th) the disease has no tendency to abate, notwithstanding a considerable fall of temperature.

